# Chemical differences in cover crop residue quality are maintained through litter decay

**DOI:** 10.1371/journal.pone.0289352

**Published:** 2023-07-27

**Authors:** Resham Thapa, Miguel Cabrera, Harry H. Schomberg, Chris Reberg-Horton, Hanna Poffenbarger, Steven B. Mirsky

**Affiliations:** 1 Department of Agricultural and Environmental Sciences, Tennessee State University, Nashville, Tennessee, United States of America; 2 Department of Crop and Soil Sciences, University of Georgia, Athens, Georgia, United States of America; 3 USDA-ARS Sustainable Agricultural Systems Laboratory, Beltsville Agricultural Research Center, Beltsville, Maryland, United States of America; 4 Department of Crop and Soil Sciences, North Carolina State University, Raleigh, North Carolina, United States of America; 5 Department of Plant and Soil Sciences, University of Kentucky, Lexington, Kentucky, United States of America; Universidad de Costa Rica, COSTA RICA

## Abstract

As plant litter decomposes, its mass exponentially decreases until it reaches a non-zero asymptote. However, decomposition rates vary considerably among litter types as a function of their overall quality (i.e., carbon:nitrogen (C:N) ratio and litter chemistry). We investigated the effects of hairy vetch (HV: *Vicia villosa* Roth):cereal rye (RYE: *Secale cereale* L.) biomass proportions with or without broadcasted poultry manure on overall litter quality before and during decomposition. As HV biomass proportions increased from 0 to 100%, the relative susceptibility of HV:RYE mixtures to microbial decomposition increased due to: (i) decrease in the initial C:N ratio (87:1 to 10:1 in 2012 and 67:1 to 9:1 in 2013), (ii) increase in the non-structural labile carbohydrates (33 to 61% across years), and (iii) decrease in the structural holo-cellulose (59 to 33% across years) and lignin (8 to 6% across years) fractions. Broadcasted poultry manure decreased the overall initial quality of HV-dominated litters and increased the overall initial quality of RYE-dominated litters. Across all HV:RYE biomass proportions with or without poultry manure, chemical changes during litter decay were related to proportional mass loss. Therefore, the relative decrease in carbohydrates and the concomitant increase in holo-cellulose and lignin fractions were more pronounced for fast decomposing litter types, i.e., litters dominated by HV rather than RYE. While our results suggest possible convergence of litter C:N ratios, initial differences in litter chemistry neither converged nor diverged. Therefore, we conclude that the initial chemistry of litter before decomposition exerts a strong control on its chemical composition throughout the decay continuum.

## Introduction

Decomposition of plant litter influences carbon (C) and nitrogen (N) cycling in natural and managed ecosystems. While climate predominantly controls plant litter decomposition at global and regional scales, litter quality prevails at a local scale [1,2]. In a recent litterbag decomposition study conducted across 105 locations in the mid-Atlantic and Southeastern US, Thapa et al. [3] developed a simple empirical model to estimate the effects of litter quality and weather on decay rates. Decomposition of plant litter was driven by C:N ratio, holo-cellulose concentrations, air relative humidity, and the number of rainy days. Many ecological studies have also shown that plant litter decomposition and associated N release rates are related to tissue N and labile carbohydrate concentrations and decrease with increasing recalcitrant structural compounds such as holo-cellulose and lignin as well as C:N and lignin:N ratios [3–6].

Moisture and temperature within the litter layer are also key drivers of plant litter decomposition in conservation tillage systems [7,8]. The ability of surface litter to retain and hold moisture during wetting (rainfall and dew) and drying (evaporation) events depends on its physical and chemical characteristics. The water retention capabilities of plant litter increase as the concentration of labile carbohydrates increases [9] and that of hydrophobic lignin concentrations decreases [8,10]. In addition, total porosity increases as holo-cellulose is degraded from the cell wall of crop residues [11], thereby causing water to be readily gained and lost with progressive decomposition [12]. Therefore, litter chemistry also exerts indirect control on decomposition via its effect on water retention properties.

Litter quality influences abundance and structure of soil microbial communities [13,14], turnover and stabilization of soil organic matter [15–17], as well as soil aggregation and soil C and nutrient cycling [18]. Litter quality (both initial differences as well as changes in litter chemistry during decomposition) also controls how C from litter inputs is primarily stored in soils: particulate organic matter vs mineral-associated organic matter [19,20]. According to Microbial Efficiency-Matrix Stabilization framework [21], higher quality litters–plant litters with lower C:N ratio but higher labile than recalcitrant compounds–may be more stabilized in mineral-associated organic matter fractions, whereas lower quality litters favor particulate organic matter formation. This is because labile plant compounds are used more efficiently by soil microbes to build microbial biomass, which is an important precursor of stable mineral-associated organic matter. On the other hand, recalcitrant structural plant compounds transform into particulate organic matter, which is a faster turnover fraction of soil organic matter that promotes stable aggregate formation. Therefore, elucidating how litters with differing functional traits differs in their initial quality and how litter quality changes over the course of decomposition will shed more light towards both short- and long-term soil organic matter formation, persistence, and functions. This in turn will assist us in better managing agroecosystems for long-term sustainability and climate change mitigation.

In managed agroecosystems, litter quality can be manipulated to improve nutrient cycling and soil carbon storage. For example, initial litter C:N ratio and chemistry varies considerably depending on crop species and mixture compositions [22,23] as well as the growth stage of mixture components [7,24,25]. In general, grasses are characterized by high C:N ratio and greater concentrations of structural compounds such as holo-cellulose as compared to labile carbohydrates [7,8,23,26]. Whereas legumes tend to have lower C:N ratio but higher carbohydrates than holo-cellulose. Moreover, both litter C:N ratio and the degree of recalcitrance increases with increased plant maturity [27]. Although legume:grass mixtures were extensively studied in the past in agricultural settings, these studies were mostly focused on determining their relative productivity (biomass, N content, and C:N ratio), their decomposability following termination, and ecosystem service provisioning [23,28,29]. Only a handful of studies assessed both C:N ratio and biochemical composition of legume:grass mixtures and found that the mixtures had intermediate levels of C:N ratio and litter chemistry between component monoculture species [23,26]. These initial differences in litter C:N ratio and chemistry among legume:grass mixtures influences overall litter decomposition and N release rates [30,31] and hence, litter-derived C inputs in soils. Furthermore, component species within mixtures could also interact such that N addition from legumes could slow down lignin breakdown in grasses while stimulating the decomposition of holo-cellulose [32–34]. Therefore, it is critical to empirically assess initial litter chemistry as a function of legume:grass biomass proportions in the mixtures. Yet, such studies are currently lacking.

Chemical complexity of decomposing litters continuously changes as a result of the relative susceptibility of different chemical constituents to leaching and microbial breakdown as well as the formation of secondary microbial byproducts [35]. Wickings et al. [36] postulated three mutually non-exclusive hypotheses to describe chemical changes during litter decomposition: the ‘chemical convergence hypothesis’ in which initial differences in chemistries among diverse litters decline through time to reach common chemistry; the ‘initial litter quality hypothesis’ in which initial differences in chemistries among diverse litters persist throughout the decay continuum; and the ‘decomposer control hypothesis’ in which the same litter, when exposed to distinct decomposer communities, becomes chemically diverse during decomposition. While studies have supported the chemical convergence hypothesis [5,35,37,38], others have not. For example, Wickings et al. [36] observed divergence in the litter chemistry of grass (primarily *Bromus inermis*, Leyss) and corn (*Zea mays* L.) residues during decomposition and suggests that the chemical changes were strongly regulated by decomposer communities’ characteristics. Wang et al. [39], on the other hand, observed the convergence of some C compounds but the divergence of other C compounds during the decomposition of high- and low-quality leaf litters in forest ecosystems. The lack of consensus among studies warrants more research to revisit these existing hypotheses regarding changes in litter chemistry during decomposition.

To investigate both initial differences in litter quality and changes in litter quality throughout the decay continuum, we conducted litter manipulation experiments by placing residues of legume and grass cover crop (CC) species at varying biomass proportions in litterbags under a suite of pelletized poultry litter (*hereafter*, *referred as ‘poultry manure’ to avoid any confusion with the use of term ‘litter’; In this paper*, *the term ‘litter’ indicate any material that was kept inside the litterbag*, *i*.*e*., *either CC residues alone or CC residues with poultry manure*) management strategies. The main objectives of this study were to: (i) assess the effects of legume-grass biomass proportions with or without poultry manure on overall initial litter quality (i.e., C:N ratio and litter chemistry before decomposition); (ii) determine changes in litter quality over the course of decomposition; and (iii) model time-invariant changes in litter chemistry over the course of decomposition.

## Materials and methods

### Field experiments

Field experiments were conducted at the USDA-ARS Beltsville Agricultural Research Center on two separate farms (Table 1): an organically managed North farm during 2012 and a conventionally managed South farm during 2013. No permits were required to conduct this field research. A detailed description of the field operations and litterbag decomposition experiments was previously provided in Poffenbarger et al. [30]. Experiments were laid out in a split-block design with CCs as the main plot and poultry manure management as the sub-plots. Five hairy vetch (HV, *Vicia villosa* Roth):cereal rye (RYE, *Secale cereale* L.) treatments were planted in a proportional replacement series in early fall of the previous year at ratios of 0:100, 20:80, 60:40, 80:20, and 100:0. Seeding rates for each species in the mixture were based on their monoculture seeding rates (HV: 34 kg ha^-1^; RYE: 168 kg ha^-1^). In the spring, four poultry manure and tillage treatments were established within each CC plots: no-poultry manure control/no-till, subsurface banded poultry manure/no-till, broadcast poultry manure/no-till, and broadcast poultry manure/tillage. Each treatment combination was replicated three times. Due to large ash concentrations (i.e., >15%), we could not determine C chemistry in litters retained from buried bags (i.e., tillage treatment). Therefore, this study only characterized chemical changes during surface litter decomposition in no-till systems.

**Table 1:** Details on initial site characteristics and field operations in Beltsville, MD, USA.

Research sites	Organically managed North Farm	Conventionally managed South Farm
Location	39.03 N, 76.93 W	39.02 N, 76.94 W
Experimental year	2012	2013
Field history	Organically managed since 2003	Conventionally managed
Soil type	Hatboro series (fine-loamy, mixed, mesic Typic Endoaquults)	Codorus series (fine-loamy, mixed, active, mesic Fluvaquentic Dystrudepts)
Bulk density, mg m^-3^	1.35	1.35
Sand, %	44	52
Clay, %	18	18
pH, 1:1 soil/water	6.7	5.7
**Field operations:**		
Cover crop planting	10/7/2011	9/25/2012
Cover crop termination	5/17/2012	5/22/2013
Corn planting	5/17/2012	5/22/2013
Pelletized poultry litter (PPL), Mg ha^-1^	3.6	3.4
Pre-plant PPL	5/17/2012	5/22/2013
Subsurface banded PPL	7/3/2012	6/25/2013
Corn harvest	9/14/2012	9/18/2013

Cover crops in no-till plots were terminated using a roller/crimper (I&J Manufacturing, Gap, PA). At the time of termination in 2012 (organically managed North Farm), HV was in full flower and RYE was in soft dough stage (Zadoks stage 85); in 2013 (conventionally managed South Farm), HV was at 50% flowering and RYE was in milk stage (Zadoks stage 75). A 105-d corn (*Zea mays* L.) was planted on the same day as CC termination (Table 1). Both subsurface banded and broadcast treatments received poultry manure (Perdue Agricycle, LLC, Seaford, DE), corresponding to 67 kg PAN ha^-1^. In the broadcast treatment, poultry manure was hand-applied on the same day just after corn planting. In the subsurface banded treatment, poultry manure was side-dressed using a subsurface banding applicator at the corn V5-V8 growth stage.

### Litterbag decomposition experiment

We tracked the proportion of initial CC residues, or ‘CC residues + poultry manure’ for the broadcast treatment, mass remaining in nylon mesh litterbags (0.30 by 0.30 m dimensions, 1 mm mesh size) over time. The targeted HV:RYE biomass proportions in the litterbags were: 0:100, 25:75, 50:50, 75:25, and 100:0. To determine the initial residue water contents and estimate corresponding fresh weights of each CC species in the litterbags, we collected aboveground biomass samples from at least two 0.5 m^2^ areas from each CC plots 5 d before termination. The samples were weighed fresh and then oven-dried at 60°C for 4 d to determine initial residue water contents. The actual HV:RYE biomass proportions, total dry biomass, and initial C:N ratios of the litterbag contents at *t*_0_ were presented in Table 2. Although the actual HV:RYE biomass proportions differed slightly from the target values, we will refer to biomass proportions in this study by the target values, unless stated otherwise.

**Table 2:** Targeted and actual hairy vetch (HV):Cereal rye (RYE) biomass proportions, total dry biomass, and C:N ratios of decomposing litters containing cover crop (CC) residues with or without poultry manure at initiation of the 2012 and 2013 litterbag decomposition studies. Total dry biomass and initial C:N ratios are presented on an ash-free basis. Standard errors are shown in parenthesis.

Year	HV:RYE biomass proportions		Litters	Total dry biomass	Initial C:N ratio
	Target	Actual			
				**kg ha** ^**-1**^	
2012	0:100	0:100	CC residues only	8242 (283)	87 (6.3)
2012	25:75	20:80	CC residues only	8019 (186)	39 (2.6)
2012	50:50	42:58	CC residues only	7542 (217)	22 (1.0)
2012	75:25	67:33	CC residues only	6828 (253)	15 (0.6)
2012	100:0	100:0	CC residues only	5888 (347)	10 (0.5)
2012	0:100	0:100	CC residues + Poultry manure	10559 (388)	33 (2.9)
2012	25:75	20:80	CC residues + Poultry manure	10122 (226)	20 (1.1)
2012	50:50	42:58	CC residues + Poultry manure	9821 (472)	18 (1.3)
2012	75:25	67:33	CC residues + Poultry manure	8919 (479)	13 (0.3)
2012	100:0	100:0	CC residues + Poultry manure	8214 (256)	10 (0.1)
2013	0:100	0:100	CC residues only	8271 (134)	61 (3.7)
2013	25:75	25:75	CC residues only	8349 (246)	27 (1.8)
2013	50:50	50:50	CC residues only	8153 (129)	17 (1.2)
2013	75:25	75:25	CC residues only	8042 (359)	12 (0.8)
2013	100:0	100:0	CC residues only	7668 (569)	9 (0.4)
2013	0:100	0:100	CC residues + Poultry manure	10486 (172)	36 (2.1)
2013	25:75	25:75	CC residues + Poultry manure	10737 (375)	22 (1.2)
2013	50:50	50:50	CC residues + Poultry manure	10768 (173)	15 (0.5)
2013	75:25	75:25	CC residues + Poultry manure	10821 (646)	13 (1.1)
2013	100:0	100:0	CC residues + Poultry manure	9930 (299)	9 (0.6)

In the same day just before CC termination using a roller/crimper, we harvested non-decomposed HV and RYE shoot biomass at soil surface from experimental border areas. Fresh CC biomass was cut to 25-cm lengths to lay flat in the litterbag. For each litter bag, cut fresh biomass materials were weighed to obtain targeted total dry biomass and species biomass proportions, mixed uniformly, and then placed in the bags. In total, three replicate sets of six litterbags were prepared for each treatment combinations. Litterbags in the broadcast treatment also received poultry manure within the bag at the same rate as applied to the entire experimental unit. This is to ensure that all litterbags in the broadcast treatments received equal amount of poultry manure and to mimic the true effect of broadcasted poultry manure on surface residue decomposition. In the subsurface banded treatment, poultry manure was placed below the soil surface and hence, surface residues did not directly interact with the poultry manure. Therefore, litterbags in the control and subsurface banded poultry manure treatments contained only CC residues litters (‘CC residues only’), whereas those in the broadcast treatment contained both CC residues and poultry manure (‘CC residues + poultry manure’). Immediately after corn planting, surface residues were hand-removed from randomly selected 0.30 by 1.8 m section areas between corn rows within each of the three no-till subplots, and the corresponding replicate set of litterbags were pinned directly on the bare soil surface using landscape staples. To avoid any potential damages to the litterbags, they were temporarily removed from the fields during subsurface banding of poultry manure and high-residue cultivation.

Litterbags were collected at six different dates: CC termination, corn emergence, three-leaf stage (V3), eight-leaf stage (V8), silking, and physiological maturity. The litterbag contents were oven-dried (65°C), weighed, ground to pass through a 1-mm sieve, and analyzed for C and N concentrations using dry combustion via Leco CHN analyzer (Leco Corporation, St. Joseph, MI). A 1-g ground subsample from each litterbag was ashed at 500°C for 4 h to correct for soil contamination. Finally, all dry weights and C and N contents in the litterbags were adjusted to an ash-free basis. A 5-g portion of ground subsample from each litterbag was shipped to the Agricultural and Environmental Services Labs at the University of Georgia (Athens, GA) to determine crude protein (CP), non-fibrous carbohydrates (NFC), acid detergent fiber (ADF), neutral detergent fiber (NDF), fat, lignin, and ash concentrations via near infra-red reflectance spectroscopy (NIRS) technique using a scanning monochromator (model 6500; FOSS NIRSystems, Silver Spring, MD) [40,41].

### Data analysis

The C chemistry of litters containing CC residues with or without poultry manure was calculated using results from the NIRS analysis as follows:

%Carbohydrates=%CP+%Fat+%NFC (1)

%Holo−cellulose=%Cellulose+%Hemi−cellulose (2)

%Cellulose=%ADF–(%Lignin+%Ash) (3)

%Hemi−cellulose=%NDF−%ADF (4)

%Lignin=%Lignin (5)

The carbohydrates, holo-cellulose, and lignin concentrations were normalized to equal 100%.

For ‘CC residues only’ litters (i.e., both control and subsurface banded poultry manure treatments), initial litter properties were similar (Table 2). Moreover, subsurface banded treatments had litter decomposition and N release patterns similar to those observed for the control treatments [30]. In our preliminary analysis, we also found that litter chemistry before and during decomposition were similar between control and subsurface banded poultry manure treatments. These results indicate that subsurface banded poultry manure was not used by microbes decomposing surface litters. Therefore, in our final analysis, we assessed the effect of poultry manure additions on litter chemistry based on whether or not poultry manure directly comprised a component of litterbag contents, i.e., ‘CC residues only’ (both control and subsurface banded) vs ‘CC residues + poultry manure’ (broadcast treatment).

First, we analyzed litter chemistry using linear regression that included the effects of actual HV:RYE biomass proportions, poultry manure amendment, collection timings, and their interactions. The slope estimates correspond to a change in concentrations of different C constituents with respect to unit change in actual HV:RYE biomass proportions. Similarly, the intercept estimates correspond to concentrations of different C constituents in RYE monoculture (i.e., when HV biomass proportions equals 0). To determine if litterbag collection timings and poultry manure amendment affected the relationship between litter chemistry and actual HV:RYE biomass proportions, slopes and intercepts of the linear models were compared using the R *emmeans* and *multcomp* packages [42].

Second, we determined time-invariant changes in litter quality during decomposition. Litter decomposition rates depend on intrinsic (soil and weather) and extrinsic or management (litter quantity and quality) factors [3]. As a result, at any given time, the same litter could exhibit differing chemical changes under different soil and weather conditions. To overcome such issues and to express litter quality along the common decay continuum, we assessed C:N ratio as well as absolute and relative concentrations of different C constituents against proportional mass loss, rather than against time. The relative proportions of different C constituents and proportional mass loss during litter decomposition were calculated as follows:

$${\left(Relative\;proportion\;of\;C_{constituent}\right)}_{t}=\frac{{\left(Absolute\;C_{constituent}\right)}_{t}}{{\left(Absolute\;C_{constituent}\right)}_{0}}$$ (6)

$${\left(Proportional\;mass\;loss\right)}_{t}=1-\frac{{RM}_{t}}{{RM}_{0}}$$ (7)

where, *C*_*constituent*_ represents concentrations of carbohydrates or holo-cellulose, or lignin C constituents in the litterbag contents, *RM*_0_ is the initial ash-free weight of litterbag contents at time, 0, and *RM*_*t*_ is the ash-free weight of remaining litterbag contents at collection time, t. The effect of targeted HV:RYE biomass proportions and poultry manure amendment on these relationships was assessed by comparing the slopes and intercepts of the linear models as described above.

## Results

### Litter C:N ratios before and during decomposition

**Fig 1:**
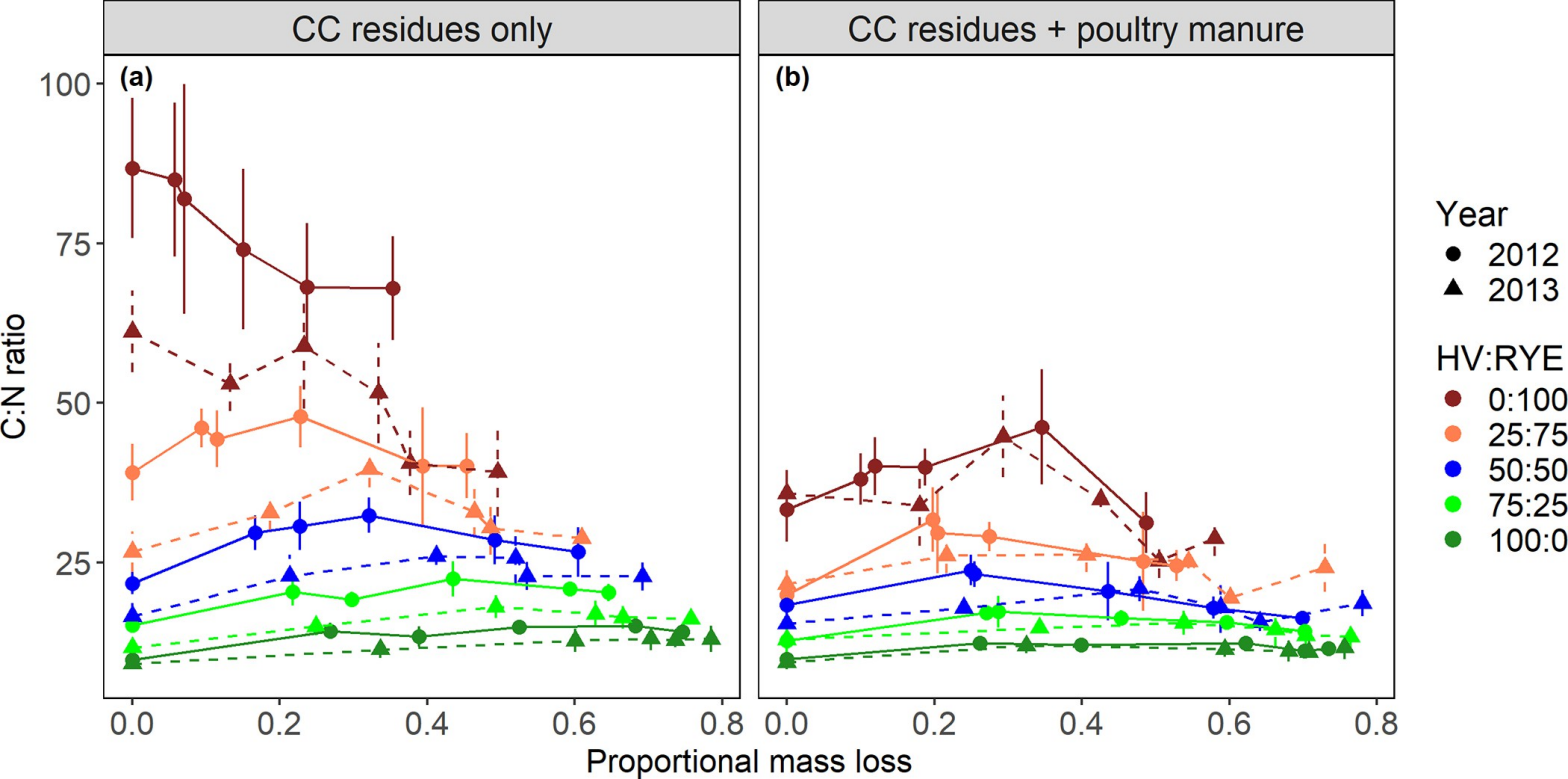
Changes in litter C:N ratios during decomposition as a function of proportional mass loss for each hairy vetch (HV):Cereal rye (RYE) biomass proportions with or without poultry manure additions. Vertical bars are ±standard deviations.

We found a significant interactive effect of year, HV:RYE biomass proportions, and poultry manure amendment on litter initial C:N ratios (p < 0.05; Fig 1). Across all HV:RYE biomass proportions except pure HV, the initial C:N ratio of litters containing CC residues alone were significantly higher in 2012 than in 2013 (p < 0.05). Whereas for litters containing both CC residues and poultry manure, the initial C:N ratios were similar between years. As the HV biomass proportion increased from 0 to 100% in the mixture, the initial C:N ratios of the litters containing CC residues alone decreased significantly from 87 for pure HV to 10 for pure RYE in 2012 and from 61 for pure HV to 9 for pure RYE in 2013 (Fig 1A). Similarly, the initial C:N ratios of the litters containing both CC residues and poultry manure decreased significantly from a value of 35 for pure RYE to 10 for pure HV (Fig 1B). Poultry manure additions significantly decreased initial C:N ratios of litter containing the 0:100, 25:75, 50:50, and 75:25 HV:RYE biomass proportions in 2012 and the 0:100 and 25:75 HV:RYE biomass proportions in 2013 (p < 0.05).

As decomposition progressed, the C:N ratios of pure RYE decreased continuously and reached a minimum value of 68 in 2012 and 39 in 2013 (Fig 1). In sharp contrast, the C:N ratio of pure HV continued to increase during decomposition and reached 13–14 by final collection date (*t*_5_). The C:N ratio of HV:RYE mixtures with or without poultry manure increased initially until the proportional mass loss reached between 0.2–0.4, after which the C:N ratio started to decrease.

### Litter chemistry before and during decomposition

**Fig 2:**
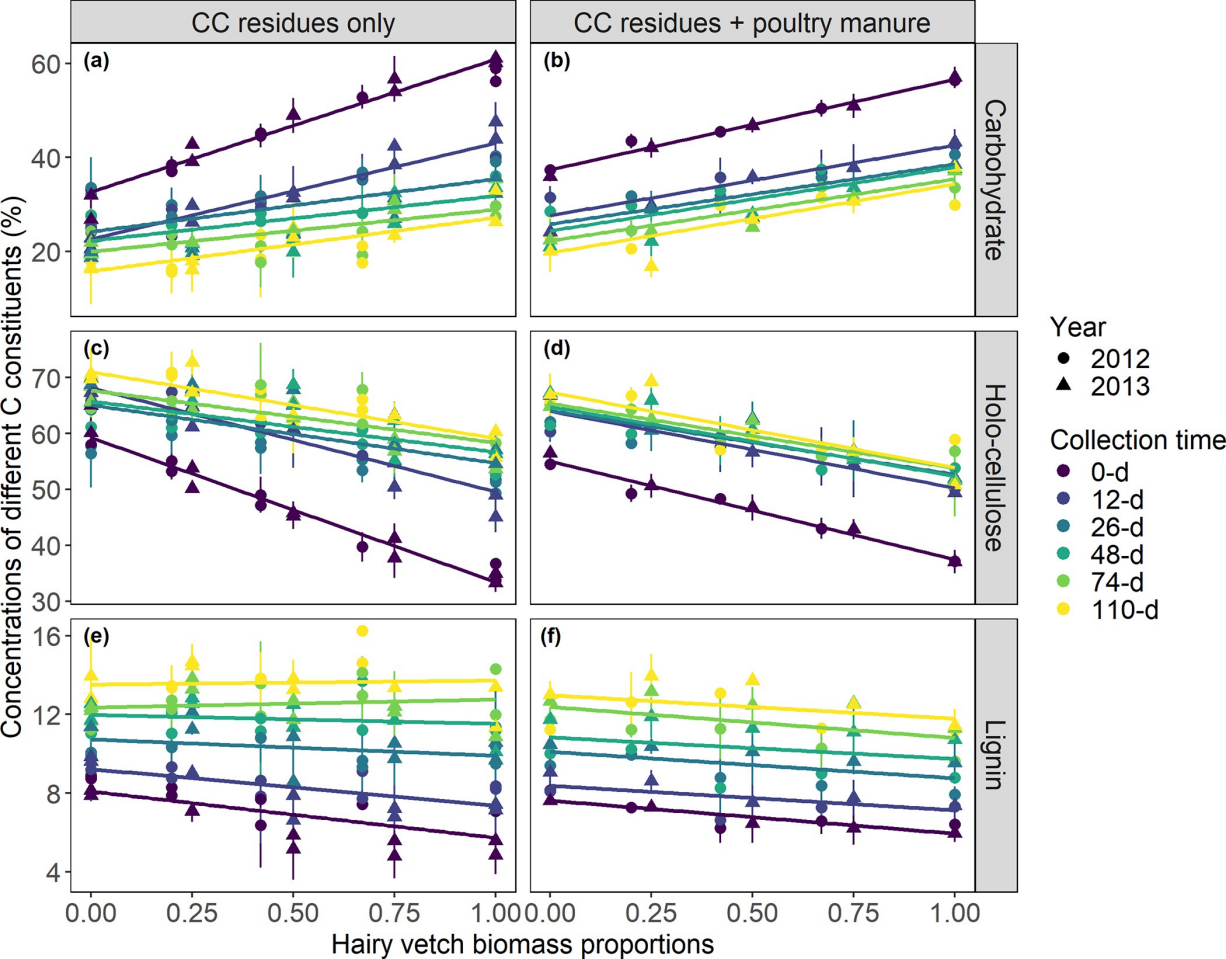
Changes in chemistry of decomposing litters containing cover crop (CC) residues with or without poultry manure as a function of actual hairy vetch (HV):Cereal rye (RYE) biomass proportions at each litterbag collection timings. Vertical bars are ±standard deviations. Parameter estimates of the linear models are presented in Table 3.

Litter chemistry was significantly affected by HV:RYE biomass proportions × poultry manure amendment × collection timings (p < 0.0001). At *t*_0_, pure RYE (Zadoks stage 75–85), on average, had 33% carbohydrates, 59% holo-cellulose, and 8% lignin. Similarly, pure HV at flowering stage had 61% carbohydrates, 33% holo-cellulose, and 6% lignin. As HV biomass proportions increased in the mixture, the carbohydrates increased linearly whereas the holo-cellulose and lignin concentrations decreased (Fig 2). Furthermore, poultry manure additions significantly affected slope and intercept estimates of these linear models. At *t*_0_, initial carbohydrate concentrations in the ‘CC residues + poultry manure’ litter had a significantly lower slope, but a significantly higher intercept estimate, than the ‘CC residues only’ litter (p < 0.05; Table 3; Fig 2A and 2B). This suggests that whilst poultry manure significantly increased initial carbohydrate concentrations in litters with high RYE proportion, it significantly decreased them in litters with high HV. In sharp contrast, poultry manure additions significantly reduced initial holo-cellulose concentrations in litters with high RYE proportion, but significantly increased in litters with high HV (p < 0.05; Table 3; Fig 2C and 2D). For initial lignin concentrations vs HV:RYE biomass proportions, the slope and intercept estimates remained unaffected due to poultry manure additions (p > 0.05; Table 3; Fig 2E and 2F).

**Table 3:** Linear model parameter estimates for carbohydrates, holo-cellulose, and lignin concentrations in the decomposing litters containing cover crop (CC) residues with or without poultry manure as a function of actual hairy vetch (HV):Cereal rye (RYE) biomass proportions at each litterbag collection timings. Standard errors are shown in parentheses.

Bag collection timing	Days after termination †	Cumulative DDD †	CC residues only ‡		CC residues + poultry manure ‡	
			Slope	Intercept	Slope	Intercept
				%		%
**Litter carbohydrate concentrations (%)**						
0	0	15	28.2 (2.1) aA	32.7 (1.2) aB	19.2 (3.0) aB	37.4 (1.8) aA
1	12	283	20.4 (2.1) abA	22.6 (1.2) bB	15.0 (3.1) aA	27.6 (1.9) bA
2	26	610	11.2 (2.0) cA	24.2 (1.2) bA	12.8 (3.0) aA	25.8 (1.8) bcA
3	48	1211	9.5 (2.2) cA	22.3 (1.3) bA	13.5 (3.0) aA	24.4 (1.8) bcA
4	74	1889	8.9 (2.2) cA	20.0 (1.3) bcA	13.1 (3.0) aA	22.3 (1.8) bcA
5	110	2780	11.4 (2.5) bcA	15.8 (1.3) cA	14.6 (3.0) aA	19.7 (1.8) cA
**Litter holo-cellulose concentrations (%)**						
0	0	15	-25.9 (1.9) aA	59.3 (1.1) cA	-17.6 (2.7) aB	55.0 (1.6) bB
1	12	283	-18.6 (1.9) abA	68.2 (1.1) abA	-13.7 (2.8) aA	64.0 (1.7) aB
2	26	610	-10.4 (1.8) cA	65.1 (1.1) bA	-11.4 (2.7) aA	64.1 (1.6) aA
3	48	1211	-9.1 (2.0) cA	65.7 (1.1) bA	-12.4 (2.7) aA	64.8 (1.6) aA
4	74	1889	-9.3 (2.0) cA	67.7 (1.1) abA	-11.6 (2.7) aA	65.4 (1.6) aA
5	110	2780	-11.9 (2.2) bcA	71.0 (1.2) aA	-13.4 (2.7) aA	67.3 (1.6) aA
**Litter lignin concentrations (%)**						
0	0	15	-2.4 (0.6) aA	8.1 (0.3) aA	-1.7 (0.8) aA	7.6 (0.5) aA
1	12	283	-1.9 (0.6) abA	9.2 (0.3) aA	*-1*.*3 (0*.*8) aA*	8.4 (0.5) abA
2	26	610	*-0*.*8 (0*.*5) abA*	10.7 (0.3) bA	-1.4 (0.8) aA	10.1 (0.5) bcA
3	48	1211	*-0*.*5 (0*.*6) abA*	12.0 (0.3) bcA	*-1*.*1 (0*.*8) aA*	10.8 (0.5) cdA
4	74	1889	*0*.*4 (0*.*6) bA*	12.3 (0.3) cdA	-1.6 (0.8) aA	12.4 (0.5) deA
5	110	2780	*0*.*2 (0*.*7) bA*	13.5 (0.3) dA	*-1*.*2 (0*.*8) aA*	13.0 (0.5) eA

† Litterbag pick-up days after termination and cumulative decomposition degree days (DDD) were averaged values across site-years. Cumulative DDD was calculated by summing mean daily temperatures above 0°C.

‡ Different lowercase letters are used to indicate significant differences between litterbag collection dates within the same variable and column (*P* < 0.05). Different uppercase letters are used to indicate significant differences between ‘CC residues only’ and ‘CC residues + poultry manure’ litters within the same variable and row (*P* < 0.05). All slope estimates are significantly different from one (P < 0.05), unless italicized.

**Fig 3:**
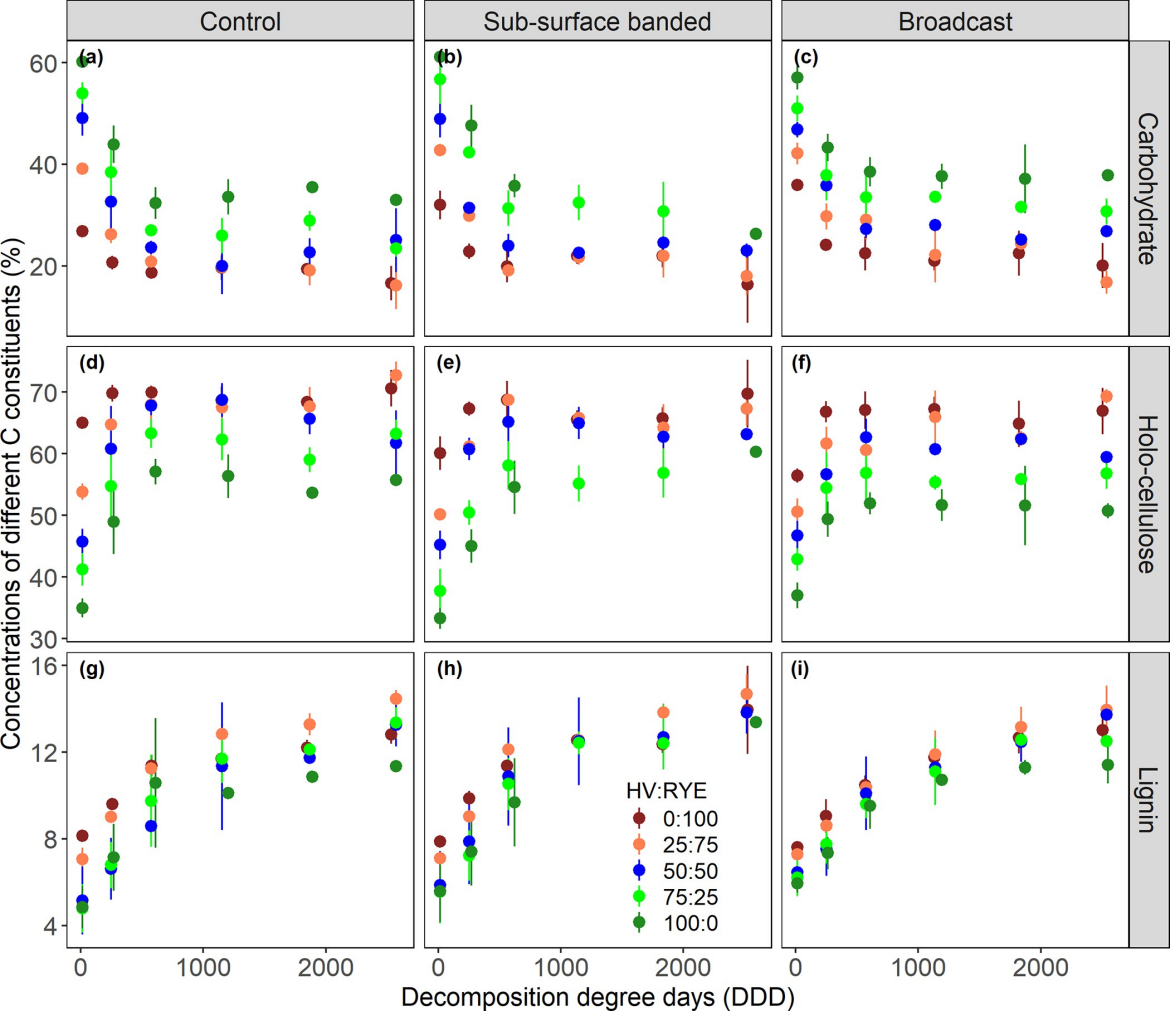
Changes in carbohydrates, holo-cellulose, and lignin concentrations in decomposing litters containing a range of hairy vetch (HV):Cereal rye (RYE) biomass proportions and subjected to different poultry manure management strategies during the 2013 corn growing season in a conventionally managed south farm at Beltsville Agricultural Research Center. Vertical bars are ±standard deviations.

As decomposition progressed, rapid shifts in litter chemistry occurred between litterbag installation and second collection date, i.e., a period of 26 days during which cumulative decomposition degree days averaged 610 (Fig 3). During this early stage of decomposition (*t*_0→2_), carbohydrates decreased whereas holo-cellulose increased rapidly across all HV:RYE biomass proportions with or without poultry manure. Moreover, the concentrations of C constituents in the ‘CC residues only’ litter were significantly affected by HV:RYE biomass proportions (Table 3; Fig 2A, 2C and 2E). We found that carbohydrates in HV-dominated litters decreased more than that in RYE-dominated litters. By *t*_2_, carbohydrates decreased by 42% for pure HV to 26% for pure RYE. Correspondingly, a larger increase in the holo-cellulose and lignin concentrations was observed in HV-dominated litters. By *t*_2_, holo-cellulose and lignin concentrations increased by 64 and 74%, respectively for pure HV to 10 and 32%, respectively for pure RYE. However, for litters containing both CC residues and poultry manure, relative shifts in litter chemistry during decomposition remained unaffected by HV:RYE biomass proportions (Table 3; Fig 2B, 2D and 2F). During later stages of decomposition (*t*_2→5_), both carbohydrates and holo-cellulose remained relatively constant for both ‘CC residues only’ and ‘CC residues + poultry manure’ litters (Fig 3A–3F). Lignin, on the other hand, increased progressively throughout the decomposition period and reached values between 13–14% after 110 days of decomposition (Fig 3G–3I).

### Time-invariant changes in litter chemistry during decomposition

**Fig 4:**
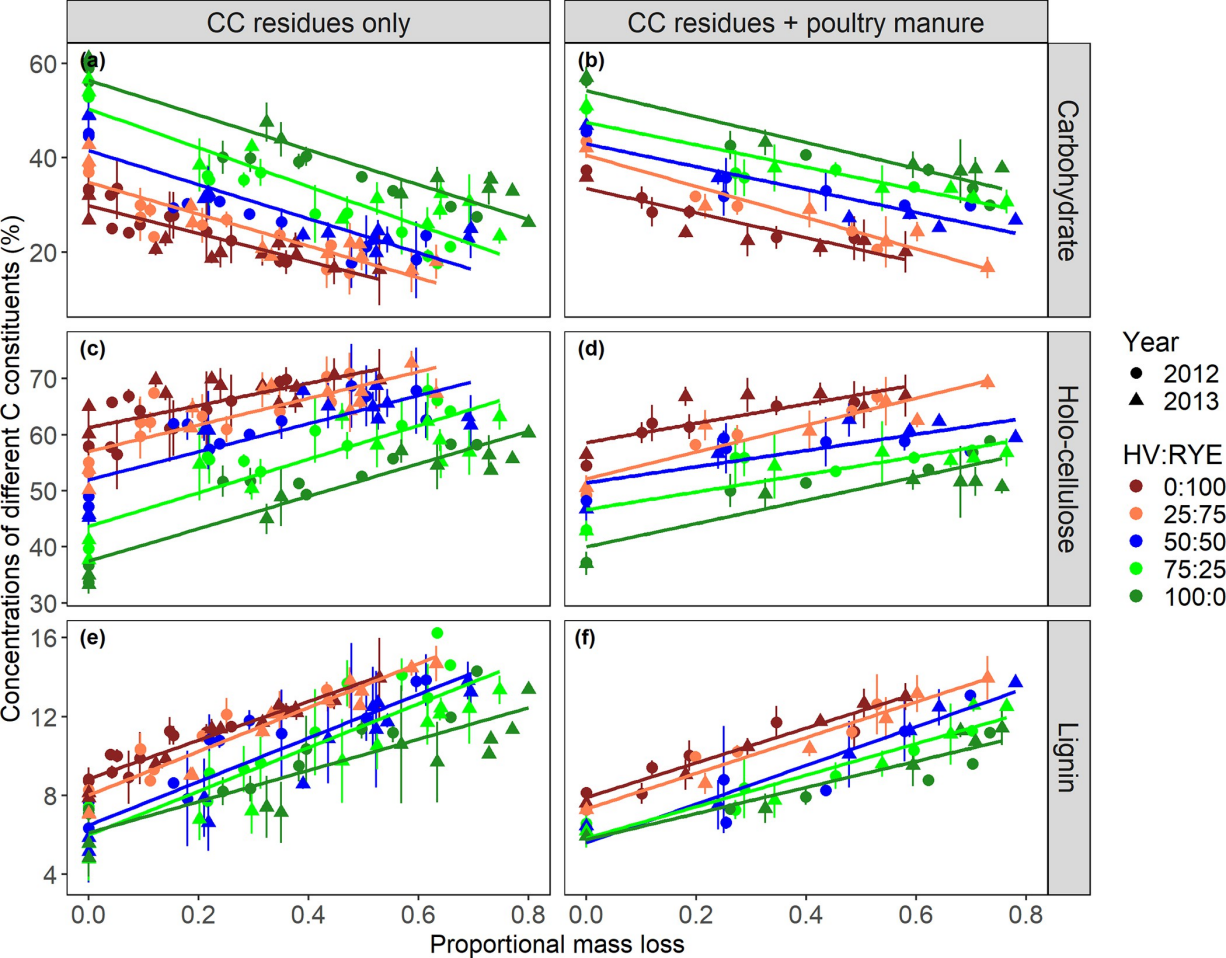
Changes in carbohydrates, holo-cellulose, and lignin concentrations in the decomposing litters containing cover crop (CC) residues with or without poultry manure as a function of proportional mass loss during decomposition for each hairy vetch (HV):Cereal rye (RYE) biomass proportions. Vertical bars are ±standard deviations. Parameter estimates of the linear models are presented in Table 4.

We related changes in litter chemistry, both in terms of absolute and relative concentrations, to proportional mass loss during decomposition. Across all HV:RYE biomass proportions, the absolute carbohydrate concentrations showed a linear decrease whereas holo-cellulose and lignin increased linearly with increasing proportional mass loss (Fig 4). The slopes of the linear relationships did not differ significantly among all HV:RYE biomass proportions (Table 4). As greater mass was lost initially from HV-dominated litters, it also resulted in a fast change in litter chemistry in proportion to litter mass loss. The intercepts of the linear relationships differed significantly among HV:RYE biomass proportions indicating differences in initial litter chemistry (Table 4). As HV:RYE biomass proportions increased, the intercepts for carbohydrates also increased proportionally, whereas the intercepts for holo-cellulose and lignin decreased.

**Table 4:** Linear model parameter estimates for carbohydrates, holo-cellulose, and lignin concentrations in the decomposing litters as a function of proportional mass loss for each targeted hairy vetch (HV):Cereal rye (RYE) biomass proportions with or without poultry manure additions. Standard errors are shown in parentheses.

HV:RYE biomass proportions	CC residues only †			CC residues + poultry manure †	
	Slope	Intercept		Slope	Intercept
		%			%
**Litter carbohydrate concentrations (%)**					
0:100	-29.6 (4.8) aA	29.9 (1.2) eA		-26.1 (5.6) aA	33.5 (1.8) cA
25:75	-33.8 (3.8) aA	34.8 (1.3) dB		-33.0 (4.8) aA	40.5 (2.1) bcA
50:50	-36.1 (3.4) aA	41.5 (1.4) cA		-24.3 (4.3) aB	43.0 (2.1) bA
75:25	-41.1 (3.2) aA	50.3 (1.5) bA		-23.7 (4.2) aB	47.5 (2.2) abA
100:0	-36.9 (3.1) aA	56.4 (1.6) aA		-27.4 (4.0) aA	54.2 (2.2) aA
**Litter holo-cellulose concentrations (%)**					
0:100	19.8 (4.7) aA	61.3 (1.2) aA		17.3 (5.4) aA	58.6 (1.8) aA
25:75	23.7 (3.7) aA	57.0 (1.3) abA		24.0 (4.7) aA	52.1 (2.0) abB
50:50	25.1 (3.3) aA	52.0 (1.4) bA		14.5 (4.1) aB	51.4 (2.0) abA
75:25	30.0 (3.1) aA	43.7 (1.4) cA		15.7 (4.1) aB	46.6 (2.1) bcA
100:0	29.0 (3.0) aA	37.5 (1.5) dA		20.8 (3.9) aA	40.0 (2.1) cA
**Litter lignin concentrations (%)**					
0:100	9.8 (1.3) aA	8.8 (0.3) aA		8.8 (1.5) aA	7.9 (0.5) aA
25:75	11.1 (1.0) aA	8.0 (0.3) aA		9.1 (1.3) aA	7.3 (0.5) abA
50:50	11.1 (0.9) aA	6.5 (0.4) bA		9.8 (1.1) aA	5.6 (0.5) bA
75:25	11.1 (0.8) aA	6.0 (0.4) bA		8.0 (1.1) aB	5.8 (0.6) bA
100:0	7.9 (0.8) aA	6.1 (0.4) bA		6.6 (1.0) aA	5.8 (0.6) bA

† Different lowercase letters are used to indicate significant differences between targeted HV:RYE biomass proportions within the same variable and column (*P* < 0.05). Different uppercase letters are used to indicate significant differences between ‘CC residues only’ and ‘CC residues + poultry manure’ litters within the same variable and row (*P* < 0.05).

For litters containing CC residues with or without poultry manure, initial differences in litter chemistry among HV:RYE biomass proportions persisted throughout the decomposition period (Fig 4). To fit a single model across all HV:RYE biomass proportions, we calculated relative proportions of different C constituents throughout the decay continuum based on their respective initial values. The relative proportions of carbohydrate concentrations decreased linearly with increasing proportional mass loss during decomposition. Concomitantly, the relative proportions of holo-cellulose and lignin concentrations increased linearly. Poultry manure additions significantly decreased the slopes of these linear relationships but had no significant effect on the intercept estimates (Table 5).

**Table 5:** Linear model parameter estimates and coefficients of determination (R^2^) for relative proportion of initial carbohydrates, holo-cellulose, and lignin concentrations in the decomposing litters as a function of proportional mass loss across all hairy vetch (HV):Cereal rye (RYE) biomass proportions with or without poultry manure additions. Standard errors are shown in parentheses.

Litters	Slope †	Intercept †	R^2^	p-value
**Relative proportion of initial carbohydrate concentrations**				
CC residues only	-0.72 (0.04) A	0.91 (0.02) A	0.76	<0.0001
CC residues + poultry manure	-0.51 (0.05) B	0.91 (0.02) A	0.71	<0.0001
**Relative proportion of initial holo-cellulose concentrations**				
CC residues only	0.76 (0.04) A	1.03 (0.02) A	0.69	<0.0001
CC residues + poultry manure	0.43 (0.06) B	1.06 (0.03) A	0.61	<0.0001
**Relative proportion of initial lignin concentrations**				
CC residues only	1.89 (0.09) A	0.95 (0.03) A	0.78	<0.0001
CC residues + poultry manure	1.32 (0.11) B	0.94 (0.05) A	0.81	<0.0001

† Different uppercase letters are used to indicate significant differences between ‘CC residues only’ and ‘CC residues + poultry manure’ litters within the same variable and column (*P* < 0.05).

## Discussion

Overall results demonstrated that: i) increasing HV:RYE biomass proportions improved initial litter quality via. decreasing C:N ratios and increasing labile carbohydrates while decreasing holo-cellulose and lignin fractions, ii) broadcasting poultry manure improved initial quality of RYE-dominated litters but not of HV-dominated litters, iii) litter C:N ratios containing some HV and/or poultry manure initially increased and then decreased indicating possible convergence at later stages of decomposition, and iv) chemical differences in initial litter chemistry are maintained throughout the decay continuum supporting the ‘initial litter quality hypothesis’.

### Initial litter quality: Effect of HV:RYE biomass proportions and broadcasted poultry manure applications

Litter C:N ratios have been frequently used to describe decomposition and N mineralization-immobilization kinetics [7,43]. Hairy vetch had a low initial C:N ratio (9 to 10) in both site-years (Fig 1). As RYE was relatively more mature in 2012 (Zadoks stage 85) than in 2013 (Zadoks stage 75), the initial C:N ratio was also significantly higher in 2012 (87) than in 2013 (61). In both years, HV:RYE mixtures had intermediate C:N ratios between monoculture species indicating moderate decomposition rates and a more balanced N release from CC mixtures to better synchronize with peak N demand from subsequent cash crop. Litter decomposition and N release rates are also strongly influenced by differences in initial litter chemistry [1,3,24]. Fresh undecomposed litters mostly contain carbohydrates and holo-cellulose ranging from 27–61 and 33–65% of the total C, respectively. As N-rich HV biomass proportions increased in the mixtures, we saw a linear increase in carbohydrates which concomitantly decreased holo-cellulose fractions (Fig 2). These results are consistent with previous studies suggesting that N-rich legume litters are characterized by higher carbohydrates than holo-cellulose and vice-versa [7,8,10,22–26]. While lignin concentration was less variable among the HV:RYE biomass proportions considered (5–9%), it significantly decreased as HV:RYE biomass proportions increased. In sharp contrast, Lawson et al. [23] observed an increase in lignin concentrations with increasing HV:RYE biomass proportions. The observed opposing effects were likely due to differences in pure RYE lignin concentrations between these studies (~8% in the present study vs 2% in Lawson et al. [23]). Just like C:N ratios, initial litter chemistry also varied between and within CC species depending on their growth stage. As plants advance to maturity, carbohydrates decrease but holo-cellulose and lignin concentrations increase. For example, carbohydrates in pure RYE decreased 53% from tillering (Zadoks stage 30) to anthesis (Zadoks stage 60), with holo-cellulose and lignin increasing by 36 and 211%, respectively [7]. Similar results were also reported for legumes such as HV and crimson clover [24,25]. Although in general there is consensus that litter quality decreases as plant maturity increases, future research efforts should determine precise changes in litter chemistries for at least the most commonly adopted CC species across vegetative growth stages. We can then reliably estimate initial litter chemistry for any legume-grass mixture using simple linear models of the following form:

$${Mixture\;C}_{constituent}=({Legume\;C}_{constituent}-{Grass\;C}_{constituent})\cdot \frac{Legume\;Biomass}{Total\;Biomass}+{Grass\;C}_{constituent}$$ (8)

where *C*_*consituent*_ represents concentrations of carbohydrates or holo-cellulose or lignin pools in grass and legume species at specific growth stages.

Broadcast application of poultry manure improved the overall quality of RYE-dominated litters. For example, poultry manure additions significantly increased carbohydrates but significantly decreased C:N ratio and recalcitrant holo-cellulose fractions in RYE-dominated litters. This pattern was completely opposite for HV-dominated litters. These results clearly explain why broadcasting poultry manure resulted in a greater cumulative proportional mass and N loss from RYE-dominated litters but decreased cumulative proportional N loss from HV-dominated litters [30].

### How does litter C:N ratio changes during decomposition?

Across all HV:RYE biomass proportions with or without poultry manure, litter C concentrations did not vary much during decomposition. Therefore, relative shifts in the C:N ratios of litters containing either CC residues alone or both CC residues and poultry manure were related to changes in total N concentrations in the decomposing materials. In a litterbag decomposition experiment conducted with 16 different plant species from Mediterranean and temperate environments, Bonanomi et al. [44] reported that litter N concentrations increased progressively causing C:N ratio to decrease during decomposition. Going further, Berg and McClaugherty [35] found that litter N concentrations increased linearly with increasing proportional mass loss. Consistent with these findings, we also observed that the C:N ratio of decomposing pure RYE litters decreased progressively over time. This trend of decreasing C:N with decomposition was likely because litter C is consumed through microbial respiration as CO_2_ into the atmosphere while N concentrations in high C:N RYE litters increased due to soil N immobilization. Pure HV litters, on the other hand, mineralized N from their tissues throughout the decomposition period causing the C:N ratio to slightly increase over time. The maximum C:N ratio of 13–14 was observed for the decomposing pure HV litters by the final bag collection date. This value is still considerably below the critical C:N ratio of 25 to 30 above which soil N is predicted to be immobilized by decomposing litters [45].

Interestingly, the C:N ratio of CC litters containing HV and/or poultry manure initially increased and then decreased. This trend was more evident for RYE-dominated mixtures. The initial increase in C:N ratio during the early stages of decomposition could be attributed to rapid loss of water-soluble N from HV and/or poultry manure through leaching losses that were not accompanied by proportional C losses. While some of the N released from HV and/or poultry manure could be temporarily immobilized by the companion RYE litters, the net observed effect was N release until 20 to 40% of the initial mass had been lost. This suggests that in conservation tillage systems where CC residues are left on the soil surface, HV and/or broadcasted poultry manure likely had a poor interaction with the companion RYE causing N to be quickly transferred into the underlying soils. Therefore, including some legume species and/or broadcasting manures in conservation tillage cropping systems could avoid early-season N stress to subsequent cash crops. At later stages, litter C:N ratios decreased progressively hinting possible convergence of C:N ratios after several years of decomposition.

### How does litter chemistry changes during decomposition?

Across all HV:RYE biomass proportions, the initial fast decay sharply decreased readily degradable carbohydrates in the first four weeks of decomposition (Fig 3). Besides microbial degradation, a substantial amount of soluble carbohydrates can be leached out as dissolved organic C during the early phase of decomposition [46]. Such litter-derived soluble compounds can be transformed or directly sorbed by silt- and clay-sized soil minerals, efficiently forming mineral-associated organic matter in soils [14,19]. The highly decomposed structural compounds would preferentially form particulate organic matter in soils [14]. Holo-cellulose also degraded simultaneously, albeit at a relatively slower rate than carbohydrates. Despite that, the decrease in carbohydrates concomitantly increased holo-cellulose as the normalized values of all three C constituents should add up to 100%. Lignin concentrations increased progressively through time, probably due to the disappearance of carbohydrates and holo-cellulose fractions and the resultant increase in the more decay-recalcitrant lignin fractions. An increasing proportion of lignin may shield remaining carbohydrates and holo-cellulose from further decay in lignocellulosic complexes. As a result, we observed no significant change in carbohydrates and holo-cellulose concentration during the later stages of decomposition (day 26–110). Our results mirror those of previous studies [8,22,35,47]. Other studies using solid state ^13^C NMR spectroscopy also reported the relative decrease of carbohydrates (O-alkyl-C: 60–90 ppm, di-O-alkyl-C: 90–110 ppm) and the relative increase of aliphatic alkyl-C (0–45 ppm; characteristic of lipid waxes, cutins, and microbial products), methoxyl-C (45–60 ppm), and aromatic C (110–160 ppm) compounds during decomposition [13,15,39,44,47].

We found that the chemical changes during litter decomposition were related to proportional mass loss (Fig 4). While carbohydrates decreased with increasing proportional mass loss across all HV:RYE biomass proportions with or without poultry manure, holo-cellulose and lignin concentrations increased linearly. The reduction in carbohydrates and the concomitant increase in holo-cellulose and lignin during decomposition were more pronounced for fast decomposing litter types, i.e., mixtures with more HV relative to RYE. These results were evident for litters containing CC residues alone (Fig 2A, 2C and 2E; Table 3). As decomposition progressed through time, a greater proportional mass loss for HV-dominated litters caused litter chemistry to shift more rapidly than that for RYE-dominated litters. Broadcasted poultry manure resulted in a more similar decomposition curves [30] and hence, chemistry of litters containing poultry manure also shifted in a similar manner among all HV:RYE biomass proportions considered (Fig 2B, 2D and 2F; Table 3). Our results further indicate that simple linear models can be fitted to estimate chemical changes in decomposing litters based on proportional mass loss and their initial chemistries. From a practical standpoint, these simple linear equations can be used to validate more complex C compartment models in which distinct C constituents in a litter are decomposed simultaneously through time.

Our results support the ‘initial litter quality hypothesis’ suggesting initial litter chemistry before decomposition exerts strong control on its chemistry throughout the decay process [36]. Across gradients of HV:RYE biomass proportions considered, the initial chemistry of litters with or without poultry manure neither converged nor diverged during decomposition when individual constituent concentrations were evaluated in response to proportional mass loss (Fig 4). This is in contrast to both the ‘chemical convergence hypothesis’ and the ‘decomposer control hypothesis’. According to the ‘chemical convergence hypothesis’, litters with different initial chemistries tend to converge towards a common chemistry during decomposition [5,38]. In contrast, Wickings et al. [36] observed that the initial chemistry of the grass litter diverged during decomposition due to management-induced differences in enzymatic activities and decomposer communities (i.e., the ‘decomposer control hypothesis’). In our study, we expected differences in enzymatic activities and decomposer communities between the organically-managed North farm (2012) and the conventionally-managed South farm (2013). Despite that, initial differences in litter chemistry among HV:RYE biomass proportions were maintained throughout the decay continuum (Fig 4; Table 4). The observed discrepancies in results could be due to the short duration of our litterbag decomposition experiments which lasted for only ~4 months, while the earlier studies were multi-year experiments (~2 to 6.5 years) [5,36,38].

Our results also provide important management implications with regard to the fate of litter-derived C inputs in soils. Because initial differences in litter chemistry are maintained over the course of decomposition, greater amount of labile carbohydrates enters the soil from high-quality legume (e.g., pure HV) litters, likely resulting in greater accumulation of microbial-derived C in mineral-associated organic matter. Whereas structural plant compounds–such as holo-cellulose and lignin–primarily enters the soil from low-quality grass (e.g., pure RYE) litters, likely resulting in greater accrual of plant-derived C in particulate organic matter. Compared to pure grass or legume monocultures, litters from legume:grass CC mixtures (e.g., HV:RYE) constitute a broad spectrum of nutrient and stoichiometric composition that enters into the soil. Therefore, legume:grass CC mixtures may have the potential to balance the contrasting effects imposed by either monoculture species on particulate organic matter vs mineral-associated organic matter formation. Although we do not have data to directly support our hypotheses, they were backed by a recent study by Zhang et al. [48] who found that litters from CC mixtures resulted in a higher concentration of plant-derived C in particulate organic matter than legume litters, and a greater concentration of microbial-derived C in mineral-associated organic matter than grass litters. Moreover, the fact that plant organic compounds with greater chemical diversity would enter the soil from CC mixtures indicates the greater accrual and stabilization efficiency of litter-derived C inputs in soils [14]. This is likely because the greater the chemical diversity, the smaller would be the energy return on investment for microbes associated with its assimilation, subsequently resulting in its preservation and soil organic C persistence [17]. Therefore, our study provides two hypotheses that needs to be further tested in future studies: (i) litter quality dictates the dominant pathway for transformation of litter-derived C inputs into soil organic matter (particulate organic matter vs mineral-associated organic matter), and (ii) litters from legume:grass CC mixtures may balance both short-lived particulate organic matter and long-lived mineral-associated organic matter formation, thereby promoting both short- and long-term soil organic C preservation and persistence.

## Conclusions

Our study clearly demonstrates that HV:RYE biomass proportions and broadcast poultry manure applications influenced overall initial litter quality and hence, overall litter decomposition rates in conservation tillage systems. As decomposition progressed, the C:N ratio of pure RYE decreased while that of pure HV increased. The C:N ratio of litters containing some HV and/or poultry manure initially increased and then decreased, indicating net N release during the early phase of decomposition. Therefore, either including some legumes and/or broadcasting manures over surface residues in conservation tillage systems is expected to avoid early-season N stress to subsequent cash crops. Moreover, the degree of recalcitrance increased with progressive decomposition, with a relative decrease in labile carbohydrates and a relative increase in structural compounds such as holo-cellulose and lignin. Chemical changes in decomposing litters were directly related to proportional mass loss. Our study further suggests that initial differences in litter chemistries among HV:RYE biomass proportions with or without poultry manure persisted throughout the decay continuum. Thus, chemically diverse organic compounds are likely entered into the soil from grass-legume CC mixtures, likely enhancing the stabilization efficiency of litter-derived C inputs in soils promoting both short- and long-term soil organic carbon persistence.

## Supporting information

S1 Raw data (XLSX)
